# The predictive value of ^18^F-FDG PET/CT habitat radiomics combined model in evaluating EGFR gene mutations in lung adenocarcinoma

**DOI:** 10.3389/fmed.2026.1868229

**Published:** 2026-06-12

**Authors:** Ruihe Lai, Dandan Sheng, Yuzhi Geng, Chongyang Ding, Qianqian Tan, Lianjun Zhao

**Affiliations:** 1 Department of Nuclear Medicine, Nanjing Drum Tower Hospital, Clinical College of Nanjing Medical University, Nanjing, China; 2 Department of Nuclear Medicine, The Second Affiliated Hospital of Nanjing Medical University, Nanjing, China; 3 Department of Nuclear Medicine, The First Affiliated Hospital of Nanjing Medical University, Nanjing, China; 4 The Comprehensive Cancer Center of Nanjing Drum Tower Hospital, Affiliated Hospital of Medical School, Nanjing University, Nanjing, China; 5 Clinical Cancer Institute of Nanjing University, Nanjing, China

**Keywords:** ^18^F-FDG PET/CT, EGFR mutations, lung adenocarcinoma, peritumoral radiomics, SHAP analysis, tumor habitat

## Abstract

**Purpose:**

This study assessed the utility of baseline ^18^F-FDG PET/CT radiomics by integrating tumor habitat analysis with both intra- and peritumoral features to predict EGFR mutation status in lung adenocarcinoma.

**Methods:**

A total of 724 patients from two centers were allocated to training, validation, and test cohorts. Peritumoral regions were delineated with 2–8 mm radial expansions using LIFEx, while tumor habitat subregions were identified via k-means clustering. Multiple machine learning algorithms were employed to develop clinical-metabolic, intratumoral, peritumoral, habitat, and combined models. Model performance was assessed using AUC, calibration curves, DCA, and DeLong tests, and SHAP analysis was applied to interpret critical predictive features.

**Results:**

In the test cohort, the combined model achieved the highest and most favorable predictive performance for EGFR mutation (AUC = 0.862, 95% CI: 0.80–0.93), followed by the habitat model (AUC = 0.831, 95% CI: 0.76–0.90). Both models significantly outperformed all other models across datasets (all *P* < 0.05). Among peritumoral models, the 6 mm expansion version demonstrated the highest AUC. SHAP analysis indicated that 16 of the 17 key features in the habitat model originated from Habitat 1 and 2 subregions, and approximately two-thirds of the top predictive features were CT-based.

**Conclusion:**

Baseline ^18^F-FDG PET/CT radiomics provides reliable prediction of EGFR mutation. Both the habitat and combined models show comparable and strong predictive performance to guide image-informed personalized treatment, while SHAP analysis enhances interpretability for clinical implementation.

## Introduction

Epidermal growth factor receptor (EGFR) mutation represents the first identified and most prevalent driver alteration in non-small cell lung cancer (NSCLC), with particularly high incidence in lung adenocarcinoma, reaching up to 50% in Asian populations (1). This molecular alteration functions as a key biomarker for guiding personalized targeted therapy in patients with adenocarcinoma. Although tissue biopsy remains the gold standard for detecting EGFR mutation (EGFRm), obtaining sufficient samples is often limited by factors such as tumor inaccessibility, patient health status, or socioeconomic constraints (2, 3). While conventional computed tomography (CT) and magnetic resonance imaging (MRI) contribute to lung cancer diagnosis, the development of dependable imaging-based approaches for predicting EGFRm status continues to represent a significant clinical challenge.

^18^F-fluorodeoxyglucose (^18^F-FDG) positron emission tomography/computed tomography (PET/CT) offers a multimodal functional molecular imaging platform capable of simultaneously assessing glucose metabolic activity and anatomical structure in tumor tissue. Through high-throughput extraction and quantitative analysis of extensive imaging features, this modality has emerged as a pivotal tool for predicting EGFRm in NSCLC (4–7).

The peritumoral region, serving as a biologically complex interface, plays a central role in processes such as angiogenesis, inflammatory microenvironment formation, and cancer stem cell generation, thereby markedly influencing tumor initiation, progression, invasion, and metastasis (8, 9). Prior studies have demonstrated that radiomic features extracted from peritumoral regions within a few millimeters carry significant clinical value for diagnosing lymph node metastasis, predicting treatment response, and evaluating prognosis (10–12). However, conventional radiomic approaches often treat tumors as homogeneous regions of interest, overlooking the nuanced intratumoral heterogeneity.

Habitat radiomics, an emerging method in medical image analysis, enables quantitative assessment and visualization of intratumoral heterogeneity through non-invasive approaches (8, 9, 13). This technique applies unsupervised clustering algorithms to group voxels with similar radiological phenotypes into distinct microenvironmental subregions based on voxel-level radiomic features, offering a more precise characterization of intrinsic tumor heterogeneity. Current evidence indicates that habitat radiomics holds substantial potential for diverse clinical applications, including tumor differential diagnosis, prediction of immunotherapy response, assessment of lymph node metastasis, and identification of genetic mutations (14–18).

Accordingly, this study developed and validated an ^18^F-FDG PET/CT multimodal model integrating tumor microenvironment, intra- and peritumoral imaging features, and clinical-metabolic parameters to predict EGFRm status in lung adenocarcinoma. Multiple machine learning models were constructed and compared within a multicenter retrospective cohort. SHAP analysis was employed to identify key predictive features and their contributions, enhancing both model interpretability and clinical applicability.

## Materials and methods

### Patient data

**FIGURE 1:**
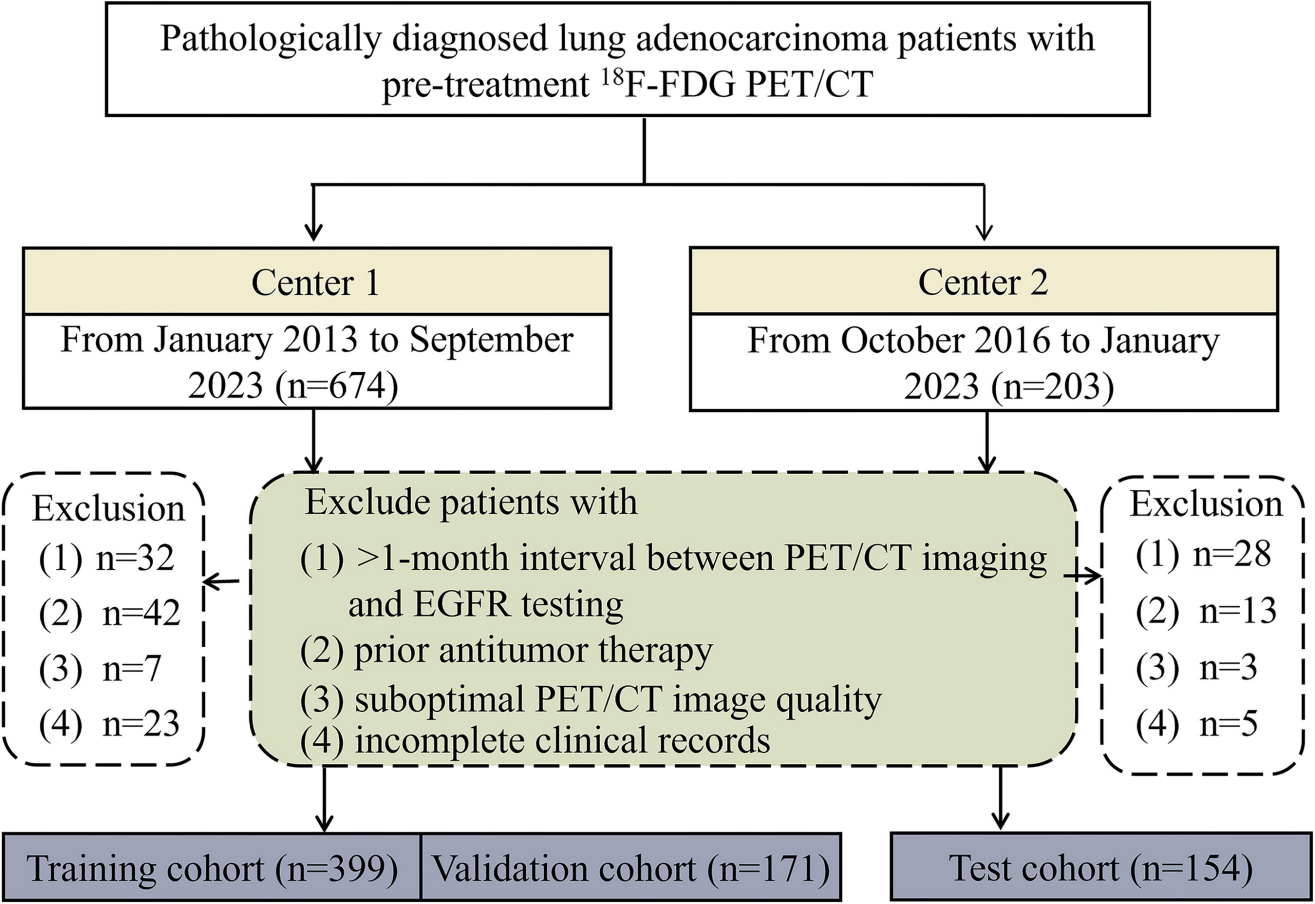
Flowchart of patient enrollment.

This retrospective study included 724 patients with histopathologically confirmed lung adenocarcinoma from two medical centers: Nanjing Drum Tower Hospital, the Affiliated Hospital of Nanjing University Medical School (*n* = 154), and Jiangsu Province Hospital, the First Affiliated Hospital of Nanjing Medical University (*n* = 570). Inclusion criteria were histopathological diagnosis of lung adenocarcinoma and available EGFR mutation status. Exclusion criteria were: (1) interval > 1 month between PET/CT imaging and EGFR testing; (2) prior antitumor therapy; (3) suboptimal PET/CT image quality; and (4) incomplete clinical records (see Figure 1 for patient selection workflow). Clinical data, including gender, age, smoking/drinking history, TNM stage, and serum tumor markers (CEA, Cyfra 21-1, NSE), were collected. The cohort comprised 355 males and 369 females, aged 27–87 years (mean, 62 years). For model development, 570 cases from the primary center were randomly assigned to a training cohort (*n* = 399) and a validation cohort (*n* = 171) at a 7:3 ratio, while the remaining 154 cases formed an external test cohort. The study was approved by the Ethics Committee of Nanjing Drum Tower Hospital (No. 2023-266-02), with a waiver of informed consent due to its retrospective design.

### EGFR mutation detection

Tissue specimens were obtained via surgical resection or CT-guided percutaneous biopsy. EGFR mutation status was assessed using a combination of next-generation sequencing (NGS) and polymerase chain reaction (PCR) analyses. Tumors harboring mutations in the specified exons were classified as EGFRm, whereas those without detectable mutations were designated as EGFR wild-type (EGFRwt).

### PET/CT scanning protocal

Whole-body ^18^F-FDG PET/CT scans were performed using two scanners: Philips GEMINI GXL and Biograph 16 PET/CT. ^18^F-FDG (radiochemical purity > 95%) was provided by Jiangyuan Andike Positron R&D. All patients fasted for at least 6 h and had blood glucose levels ≤ 11.1 mmol/L prior to intravenous administration of 3.70 MBq/kg ^18^F-FDG, followed by a 1-h uptake period before imaging. CT scans were acquired with standard parameters: 120 kV, 120 mA, 5.0 mm slice thickness, and a 144 × 144 matrix. PET data were acquired in 3D mode with corresponding bed positions and acquisition times, and CT-based attenuation correction was applied. Technical parameters for the different PET/CT models are summarized in Supplementary Table 1.

### PET/CT image preprocessing

Standardized preprocessing procedures were implemented to ensure robust, consistent, and reproducible radiomic feature extraction. All images were resampled to isotropic voxels of 1 mm^3^ to harmonize spatial resolution across different scanners and acquisition protocols. PET images underwent intensity normalization by rescaling voxel intensities to a fixed range of [0, 6000], whereas CT images were standardized using a fixed window setting (window width: 1,500 HU; window level: −500 HU), corresponding to an intensity range of [−950, 350]. These modality-specific preprocessing and normalization strategies were designed to reduce inter-scanner variability arising from differences in acquisition parameters and to enhance the stability and comparability of the extracted radiomic features.

### Volume of interest (VOI) segmentation

The PET VOIs were semi-automatically delineated using LIFEx software (version 7.5.0) (19) by a blinded, board-certified nuclear medicine physician. Initial VOIs were manually contoured slice by slice, followed by automated boundary refinement using an absolute SUV threshold of 2.5. Quantitative parameters, including SUV_*max*_, metabolic tumor volume (MTV), and total lesion glycolysis (TLG), were calculated using a relative threshold of 40% SUV_*max*_. Peritumoral regions were automatically generated in LIFEx as concentric annular VOIs with radial expansions of 2, 4, 6, and 8 mm from the tumor margin. Non-pulmonary tissues within these regions were manually excluded to ensure anatomical specificity.

**FIGURE 2:**
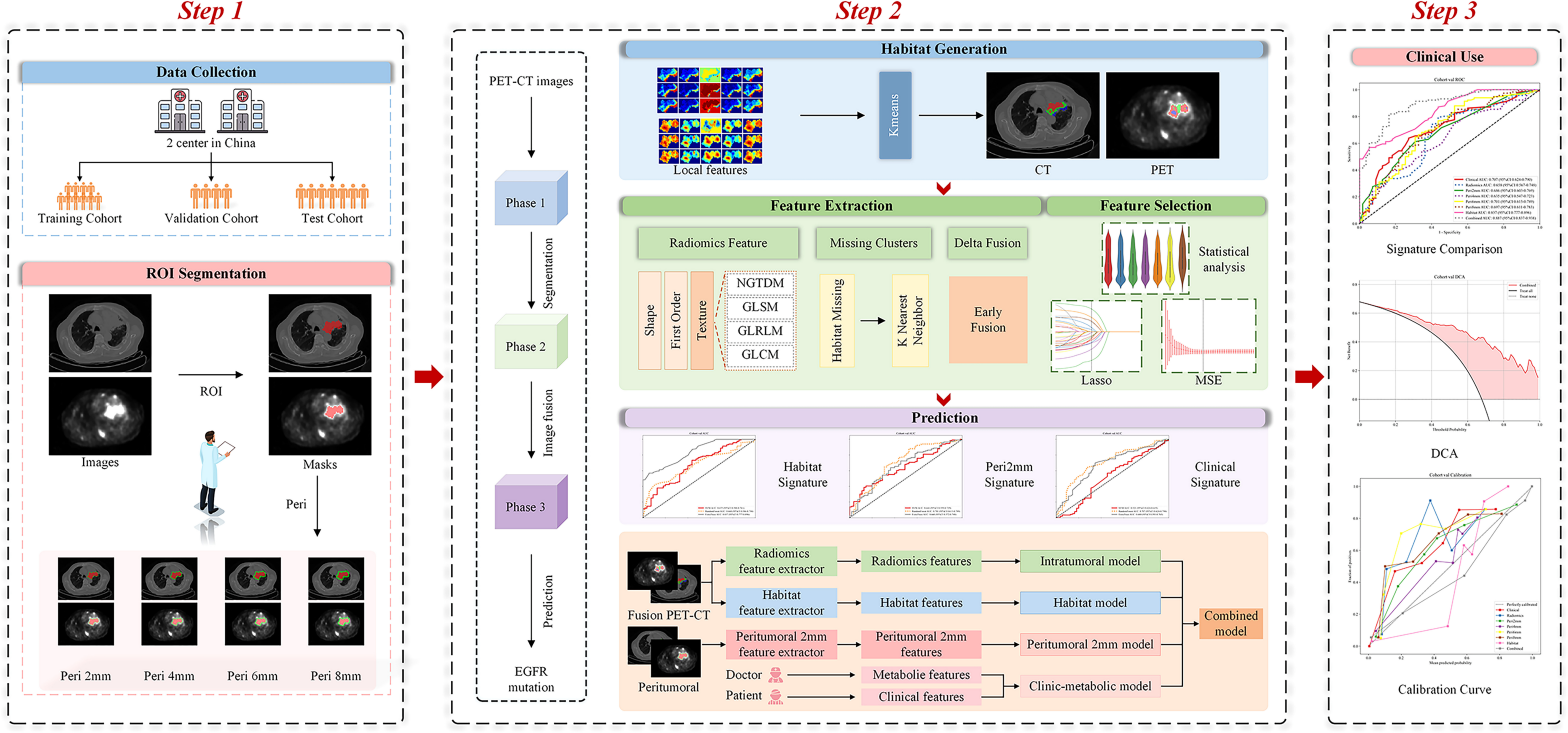
Flowchart of radiomics workflow.

For CT analysis, VOIs were independently segmented manually by two board-certified radiologists using ITK-SNAP software (version 3.8.0).^1^ The entire primary tumor volume was delineated while carefully excluding adjacent anatomical structures such as cavitary regions, bronchial trees, and vascular components. Morphological characteristics were systematically recorded, and any inter-observer discrepancies were resolved through consensus review. Inter-rater reliability was quantitatively assessed using the Dice similarity coefficient (DSC) on a randomly selected subset of 50 cases. The complete radiomic analysis pipeline is illustrated in Figure 2.

### Habitat local feature characterization and clustering

Voxel-level analysis was employed to characterize local tumor heterogeneity. A 5 × 5 × 5 sliding voxel window was applied within tumor VOIs to extract voxel-wise intensity and texture features, constructing a 39-dimensional vector encompassing first-order statistics and high-order GLCM/GLSZM parameters, as presented in Supplementary Table 2. Tumor subregions were segmented using k-means clustering, with the optimal number of clusters determined by the Calinski–Harabasz (CH) index to balance intra-cluster compactness and inter-cluster separability. This standardized feature extraction and subregion partitioning procedure was applied uniformly to both PET and CT images.

### Feature extraction and selection

Radiomics features, including morphological, intensity, and texture characteristics (GLCM, GLSZM, GLRLM, and NGTDM), were systematically extracted at both global whole-tumor and subregional habitat/peritumoral annular scales to comprehensively quantify tumor heterogeneity. To mitigate boundary artifacts and maintain spatial coherence, a K-nearest neighbor relabeling strategy was implemented. All feature extraction procedures were performed using PyRadiomics 3.0.1 in strict accordance with the Imaging Biomarker Standardization Initiative (IBSI) guidelines (20) to ensure standardized processing. Feature selection procedures were conducted exclusively in the training cohort.

PET and CT multimodal features were normalized using Z-scores, followed by refinement through a multi-stage selection pipeline: independent samples *t*-test filtering (*p* < 0.05), removal of highly correlated features (*r* > 0.9), minimum redundancy maximum relevance (mRMR) screening to balance predictive performance and redundancy, and final optimization via LASSO regression with 10-fold cross-validation to determine the optimal λ by minimizing mean squared error (MSE), resulting in a concise and discriminative feature subset for predictive modeling.

### Model construction and evaluation

All models followed an identical feature selection pipeline. Radiomics models were constructed using Support Vector Machine (SVM), Random Forest (RF), and ExtraTrees algorithms. Five model types were established: (1) Intratumoral model, based on primary tumor features as the baseline; (2) Peritumoral models, constructed for 2, 4, 6, and 8 mm annular regions to assess peripheral spatial gradient value; (3) Habitat model, derived from tumor microenvironment subregional features to capture intratumoral heterogeneity; (4) Clinical-metabolic model, built using significant variables identified through univariate and multivariate logistic regression; (5) Combined model, integrating the optimal features from the above models and visualized with a nomogram.

Model performance was evaluated using receiver operating characteristic (ROC) curves, with area under the curve (AUC), sensitivity, and specificity as the primary metrics. Calibration curves were applied to verify the agreement between predicted probabilities and actual outcomes, ensuring clinical reliability. Decision curve analysis (DCA) further quantified the net clinical benefit across threshold probabilities to assess practical applicability.

### SHAP interpretation and visualization analysis

SHAP (SHapley Additive exPlanations), a game theory-based interpretability method, was employed to quantify individual feature contributions and enhance model transparency (21). The SHAP summary plot illustrates global feature importance and directional effects through standardized SHAP values (22). For local interpretation, force plots visualize the direction and magnitude of feature impacts for individual samples (23, 24), while waterfall plots display the sequential contributions of features from baseline to final prediction (25).

### Statistical analysis

Statistical analyses were conducted using SPSS 27.0 (IBM Corp., Armonk, NY, USA) and Python (version 3.7.12). Continuous variables with normal distribution were reported as mean ± standard deviation (x̄ ± SD), whereas non-normally distributed data were expressed as median (interquartile range) [M (Q1, Q3)]. Intergroup comparisons of clinical and PET parameters were performed using the chi-square test or Mann–Whitney U test, as appropriate. Significant clinical variables and PET metabolic parameters were identified through univariate and multivariate logistic regression analyses. Radiomic feature extraction was carried out using PyRadiomics (version 3.0.1), and machine learning algorithms were implemented via the scikit-learn library (version 1.0.2). Statistical modeling was performed using statsmodels (version 0.13.2). A two-tailed *p*-value < 0.05 was considered statistically significant for all analyses.

## Results

### Patient data

Table 1 summarizes the clinical characteristics and PET/CT parameters of patients from the two participating centers, encompassing 570 subjects from Center 1 and 154 from Center 2. Notable intergroup differences were observed in Cyfra 21-1 levels, NSE levels, TNM staging, and the presence of spiculation and lobulation signs (*P* < 0.05).

**TABLE 1:** Characteristics of the study population.

Characteristics	Overall (*n* = 724)	Center 1 (*n* = 570)	Center 2 (*n* = 154)	Statistical value	*P*-value
Gender					
Male	355 (49.03)	277 (48.60)	78 (50.65)	0.20b	0.651
Female	369 (50.97)	293 (51.40)	76 (49.35)		
Age (years)^#^	64.00 (56.00, 69.00)	63.00 (56.00, 69.00)	64.00 (57.00, 69.00)	−1.19a	0.235
Smoking history					
No	507 (70.03)	396 (69.47)	111 (72.08)	0.39b	0.531
Yes	217 (29.97)	174 (30.53)	43 (27.92)		
Drinking history					
No	607 (83.84)	472 (82.81)	135 (87.66)	2.11b	0.146
Yes	117 (16.16)	98 (17.19)	19 (12.34)		
CEA (ng/ml)					
Normal	352 (48.62)	269 (47.19)	83 (53.90)	2.18b	0.140
Elevated	372 (51.38)	301 (52.81)	71 (46.10)		
Cyfra 21-1 (ng/ml)					
Normal	449 (62.02)	367 (64.39)	82 (53.25)	6.39b	0.011
Elevated	275 (37.98)	203 (35.61)	72 (46.75)		
NSE (ng/ml)					
Normal	380 (52.49)	277 (48.60)	103 (66.88)	16.26b	< 0.001
Elevated	344 (47.51)	293 (51.40)	51 (33.12)		
TNM stage					
Stage I	200 (27.62)	179 (31.40)	21 (13.64)	28.75b	< 0.001
Stage II	77 (10.64)	65 (11.40)	12 (7.79)		
Stage III	150 (20.72)	118 (20.70)	32 (20.78)		
Stage IV	297 (41.02)	208 (36.49)	89 (57.79)		
Treatment modality					
Single treatment	364 (50.28)	276 (48.42)	88 (57.14)	3.69b	0.055
Combined treatment	360 (49.72)	294 (51.58)	66 (42.86)		
Tumor location					
Peripheral type	677 (93.51)	532 (93.33)	145 (94.16)	–c	0.885
Central type	47 (6.49)	38 (6.67)	9 (5.84)		
Tumor distribution					
Right upper lobe	217 (29.97)	173 (30.35)	44 (28.57)	8.36b	0.079
Right middle lobe	64 (8.84)	56 (9.82)	8 (5.19)		
Right lower lobe	116 (16.02)	95 (16.67)	21 (13.64)		
Left upper lobe	213 (29.42)	155 (27.19)	58 (37.66)		
Left lower lobe	114 (15.75)	91 (15.96)	23 (14.94)		
Spiculation sign					
Absent	467 (64.50)	405 (71.05)	62 (40.26)	50.21b	< 0.001
Present	257 (35.50)	165 (28.95)	92 (59.74)		
Lobulation sign					
Absent	441 (60.91)	360 (63.16)	81 (52.60)	5.68b	0.017
Present	283 (39.09)	210 (36.84)	73 (47.40)		
Vacuole sign					
Absent	688 (95.03)	542 (95.09)	146 (94.81)	0.02b	0.886
Present	36 (4.97)	28 (4.91)	8 (5.19)		
Long diameter (cm)^#^	2.95 (2.10, 3.90)	2.90 (2.10, 3.90)	3.00 (2.10, 4.00)	−0.54a	0.590
Short diameter (cm)^#^	2.20 (1.60, 3.00)	2.20 (1.60, 3.00)	2.50 (1.70, 3.18)	−0.70a	0.089
SUV_*max*_^#^	10.40 (6.60, 14.30)	10.50 (6.62, 14.80)	10.00 (6.23, 12.70)	−2.39a	0.052
MTV (cm^3^)^#^	6.92 (3.25, 15.36)	6.37 (2.98, 14.53)	8.48 (4.24, 17.89)	−1.94a	0.261
TLG (g)^#^	38.00 (13.55, 110.00)	36.50 (13.00, 104.00)	44.00 (18.00, 110.75)	−1.12a	0.651

#Median (Q1, Q3); a: Mann–Whitney test with statistic reported as Z value; b: χ^2^ test with statistic reported as χ^2^ value; c: rank-sum test. CEA, carcinoembryonic antigen; Cyfra 21-1, cytokeratin 19 fragment; NSE, neuron-specific enolase; MTV, metabolic tumor volume; TLG, total lesion glycolysis.

As presented in Table 2, patients with EGFRm exhibited distinctive demographic and clinical profiles, characterized by a predominance of female sex, younger age, and non-smoking/non-drinking status. Relative to EGFRwt patients, EGFRm cases showed a significantly higher frequency of stage IV disease (44.31% vs. 33.18%, *P* = 0.025) and were more commonly associated with spiculation (38.43% vs. 28.50%, *P* = 0.011) and lobulation (41.76% vs. 32.71%, *P* = 0.023) on imaging. Moreover, EGFRm patients exhibited notably lower SUV_*max*_ (9.80 vs. 11.55, *P* < 0.001) and TLG values (33.50 vs. 52.00, *P* = 0.003) compared with their EGFRwt counterparts.

**TABLE 2:** Comparison of clinical characteristics and PET/CT parameters between EGFR wild-type and EGFR mutation patients.

Characteristics	EGFR-wt (*n* = 214)	EGFR-m (*n* = 510)	Statistical value	*P*-value
Gender				
Male	140 (65.42)	215 (42.16)	32.65b	< 0.001
Female	74 (34.58)	295 (57.84)		
Age (years)^#^	65.00 (56.00, 69.00)	63.00 (55.00, 69.00)	−2.30a	0.022
Smoking history				
No	116 (54.21)	391 (76.67)	36.23b	< 0.001
Yes	98 (45.79)	119 (23.33)		
Drinking history				
No	160 (74.77)	447 (87.65)	18.46b	< 0.001
Yes	54 (25.23)	63 (12.35)		
CEA (ng/mL)				
Normal	110 (51.40)	242 (47.45)	0.94b	0.332
Elevated	104 (48.60)	268 (52.55)		
CYFRA 21-1 (ng/mL)				
Normal	123 (57.48)	326 (63.92)	2.66b	0.103
Elevated	91 (42.52)	184 (36.08)		
NSE (ng/mL)				
Normal	115 (53.74)	265 (51.96)	0.19b	0.662
Elevated	99 (46.26)	245 (48.04)		
TNM stage				
Stage I	66 (30.84)	134 (26.27)	9.37b	0.025
Stage II	22 (10.28)	55 (10.78)		
Stage III	55 (25.70)	95 (18.63)		
Stage IV	71 (33.18)	226 (44.31)		
Treatment modality				
Single treatment	117 (54.67)	247 (48.43)	2.35b	0.125
Combined treatment	97 (45.33)	263 (51.57)		
Tumor location				
Peripheral type	198 (92.52)	479 (93.92)	0.49c	0.486
Central type	16 (7.48)	31 (6.08)		
Tumor distribution				
Right upper lobe	65 (30.37)	152 (29.80)	0.09b	0.999
Right middle lobe	18 (8.41)	46 (9.02)		
Right lower lobe	34 (15.89)	82 (16.08)		
Left upper lobe	63 (29.44)	150 (29.41)		
Left lower lobe	34 (15.89)	80 (15.69)		
Spiculation sign				
Absent	153 (71.50)	314 (61.57)	6.49b	0.011
Present	61 (28.50)	196 (38.43)		
Lobulation sign				
Absent	144 (67.29)	297 (58.24)	5.19b	0.023
Present	70 (32.71)	213 (41.76)		
Vacuole sign				
Absent	204 (95.33)	484 (94.90)	0.06b	0.810
Present	10 (4.67)	26 (5.10)		
Long diameter (cm)^#^	3.00 (2.12, 4.30)	2.90 (2.00, 3.80)	−1.35a	0.178
Short diameter (cm)^#^	2.30 (1.63, 3.20)	2.20 (1.60, 3.00)	−1.01a	0.314
SUV_*max*_^#^	11.55 (8.12, 16.17)	9.80 (5.90, 13.40)	−4.70a	< 0.001
MTV (cm^3^)^#^	7.61 (2.99, 19.13)	6.59 (3.30, 13.79)	−1.43a	0.153
TLG (g)^#^	52.00 (16.25, 166.75)	33.50 (13.00, 94.75)	−2.94a	0.003

#Median (Q1, Q3); a: Mann–Whitney test with statistic reported as Z value; b: χ^2^ test with statistic reported as χ^2^ value; c: rank-sum test. EGFRm, EGFR-mutation; EGFRwt, EGFR wild-type; CEA, carcinoembryonic antigen; Cyfra 21-1, cytokeratin 19 fragment; NSE, neuron-specific enolase; MTV, metabolic tumor volume; TLG, total lesion glycolysis.

### Univariate and multivariate logistic regression analyses

Univariate logistic regression analysis within the training cohort revealed significant correlations between EGFRm status and multiple variables, including gender, age, serum tumor markers (CEA, Cyfra 21-1, NSE), TNM stage, treatment modality, primary lesion characteristics (distribution, spiculation, lobulation, vacuole sign), lesion dimensions (maximum and minimum diameters), and metabolic parameters (SUV_*max*_, MTV). Subsequent multivariate logistic regression analysis identified gender, Cyfra 21-1 levels, TNM stage, treatment modality, and SUV_*max*_ as independent predictors of EGFRm status (Table 3). Leveraging these significant variables, an integrated clinical-metabolic model was subsequently constructed.

**TABLE 3:** Univariate and multivariate logistic regression analysis of clinical and PET/CT parameters in the training cohort.

Characteristics	Univariate analysis			Multivariate analysis		
	OR	95% CI	*P*-value	OR	95% CI	*P*-value
Gender, male/female	3.52	2.68–4.64	< 0.001	2.71	1.83–4.02	< 0.001
Age (years)	1.01	1.01–1.02	< 0.001	0.99	0.98–1.00	0.065
Smoking history, no/yes	1.19	0.88–1.61	0.34	–	–	–
Drinking history, no/yes	1.06	0.71–1.60	0.81	–	–	–
CEA (ng/mL), normal/elevated	2.49	1.94–3.20	< 0.001	1.20	0.78–1.83	0.493
CYFRA 21-1 (ng/mL), normal/elevated	1.67	1.25–2.23	< 0.001	0.56	0.36–0.88	0.034
NSE (ng/mL), normal/elevated	2.39	1.86–3.08	< 0.001	1.40	0.94–2.09	0.167
TNM stage, stage I/II/III/IV	1.32	1.24–1.41	< 0.001	1.28	1.07–1.52	0.023
Treatment modality, single/combined	2.90	2.23–3.79	< 0.001	1.64	1.11–2.44	0.038
Tumor location, peripheral/central	1.80	0.94–3.45	0.136	–	–	–
Tumor distribution#	1.24	1.18–1.32	< 0.001	0.97	0.86–1.10	0.676
Spiculation sign, absent/present	2.83	2.00–4.02	< 0.001	1.64	1.03–2.60	0.078
Lobulation sign, absent/present	2.72	2.00–3.70	< 0.001	1.32	0.87–1.99	0.279
Vacuole sign, absent/present	2.80	1.19–6.60	0.048	1.65	0.63–4.35	0.396
Long diameter (cm)	1.22	1.16–1.28	< 0.001	1.17	0.90–1.52	0.334
Short diameter (cm)	1.29	1.21–1.38	< 0.001	1.18	0.85–1.63	0.409
SUV_*max*_	1.04	1.03–1.05	< 0.001	0.94	0.91–0.97	0.001
MTV (cm^3^)	1.32	1.24–1.41	0.024	1.28	1.07–1.52	0.106
TLG (g)	1.00	1.00–1.00	0.743	–	–	–

#Right upper lobe, right middle lobe, right lower lobe, left upper lobe, left lower lobe; CEA, carcinoembryonic antigen; Cyfra 21-1, cytokeratin 19 fragment; NSE, neuron-specific enolase; MTV, metabolic tumor volume; TLG, total lesion glycolysis.

### Interobserver consistency of VOIs

Interobserver consistency was evaluated on VOI masks delineated by two investigators across 50 cases. The mean DSC value between groups was 0.969 (see Supplementary Table 3).

### Habitat local characterization and clustering

**FIGURE 3:**
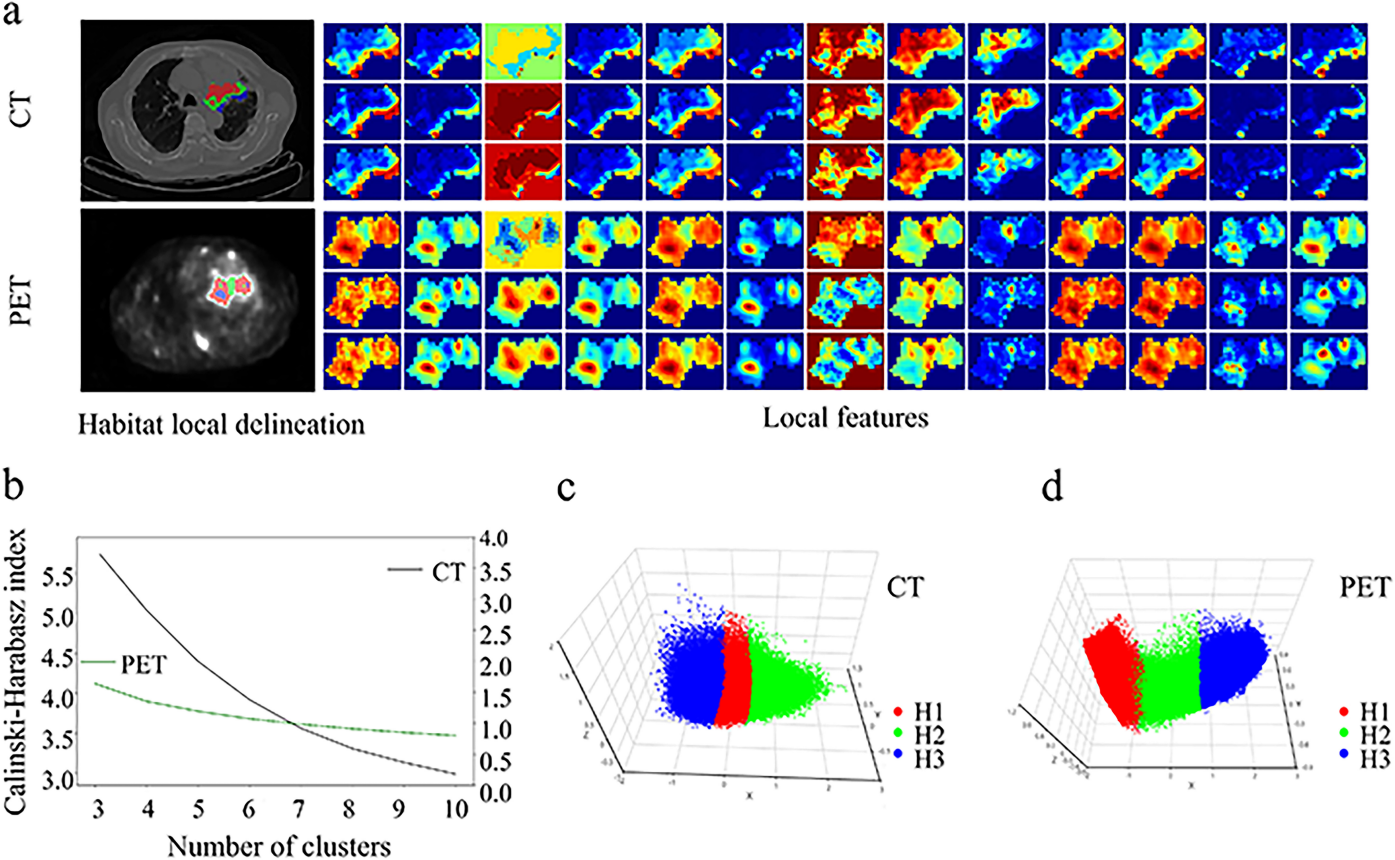
Spatial distributions of the 39 standard parameters for local habitat characterization, and visualization of tumor habitat clustering results. **(a)** Spatial distribution of the 39 standard parameters; **(b)** Variation in the CH index across different cluster numbers; **(c,d)** Representative segmentation results showing the division of the tumor region into three spatially distinct subregions based on the optimal cluster number of 3.

The study further identified 39 distinct radiomic features for intratumoral habitat characterization derived from PET/CT VOIs, as detailed in Supplementary Table 2. Corresponding spatial habitat characterization maps are shown in Figure 3.

The optimal number of clusters was determined through evaluation of clustering performance using the CH index. Across the assessed range of 3 to 10 clusters, the CH indices for both CT and PET images gradually declined with increasing cluster numbers, indicating potential over-segmentation. Accordingly, three clusters were established as the optimal configuration for tumor habitat segmentation in this study, designated as sub-Habitat 1 (H1), sub-Habitat 2 (H2), and sub-Habitat 3 (H3) (Figure 3).

### Feature selection

Feature selection was conducted using two parallel analytical strategies while preserving methodological consistency to ensure the reliability and comparability of results.

First, habitat-based feature selection was performed: A total of 1,834 PET radiomic features and 1,564 CT radiomic features were extracted from three distinct habitat subregions (H1, H2, and H3). Multimodal PET/CT features were then generated through dimensional fusion, resulting in an aggregate of 10,458 radiomic features (3 subregions × [1,834 PET features + 1,564 CT features]). Following standardized preprocessing and rigorous feature selection, 17 optimal features were ultimately identified to construct the radiomics habitat model (specific features F1–F17 are provided in Supplementary Figure 1).

Second, feature selection was applied to intratumoral and peritumoral regions: A total of 1,834 PET-derived and 1,564 CT-derived radiomic features were extracted from both the intratumoral region and multiple peritumoral regions (2, 4, 6, and 8 mm). Using a screening strategy consistent with that applied in the habitat analysis, final feature sets were established for each region, comprising 17, 5, 2, 16, and 24 features for the intratumoral area and peritumoral regions of 2, 4, 6, and 8 mm, respectively (Supplementary Figure 2).

### Optimal machine learning algorithms for predicting EGFR gene mutation across different models

**FIGURE 4:**
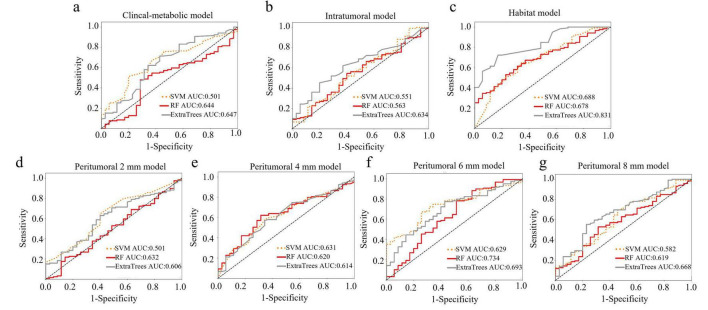
Combined ROC curves of EGFR mutation prediction models in the test set, including clinical-metabolic **(a)**, intratumoral **(b)**, different peritumoral region **(c-f)**, and habitat **(g)** models constructed by three machine learning algorithms. SVM, Support Vector Machine; RF, Random Forest; ExtraTrees, extremely randomized trees.

Within the clinical-metabolic model, the RF algorithm demonstrated superior performance during internal validation, achieving an AUC of 0.707 (95% confidence interval [CI]: 0.624–0.790). For the intratumoral and habitat models, the ExtraTrees algorithm produced optimal results, with validation cohort AUCs of 0.658 (95% CI: 0.570–0.750) and 0.837 (95% CI: 0.780–0.900), respectively. These outcomes were further corroborated in the test cohort, which yielded AUCs of 0.664 (95% CI: 0.570–0.760), 0.634 (95% CI: 0.540–0.730), and 0.831 (95% CI: 0.760–0.900) for the clinical-metabolic, intratumoral, and habitat models, respectively. Notably, the habitat model achieved the highest diagnostic accuracy (accuracy = 0.747, sensitivity = 0.723, specificity = 0.829) (Figures 4a–c and Supplementary Table 4).

For peritumoral models at varying margins, the RF algorithm was identified as optimal for the 2, 6, and 8 mm regions, with validation cohort AUCs of 0.686 (95% CI: 0.60–0.77), 0.701 (95% CI: 0.61–0.79), and 0.697 (95% CI: 0.61–0.78), respectively. In contrast, the 4 mm peritumoral model was best fitted by the ExtraTrees algorithm (AUC = 0.63, 95% CI: 0.55–0.72). Test cohort evaluation revealed AUCs of 0.632 (95% CI: 0.53–0.74), 0.614 (95% CI: 0.51–0.72), 0.734 (95% CI: 0.65–0.82), and 0.619 (95% CI: 0.51–0.73) for the 2, 4, 6, and 8 mm peritumoral models, respectively. Among these, the 6 mm peritumoral model demonstrated the highest predictive performance (Figures 4d–g and Supplementary Table 5).

### Combined model construction and comparative evaluation of predictive performance of different models

**FIGURE 5:**
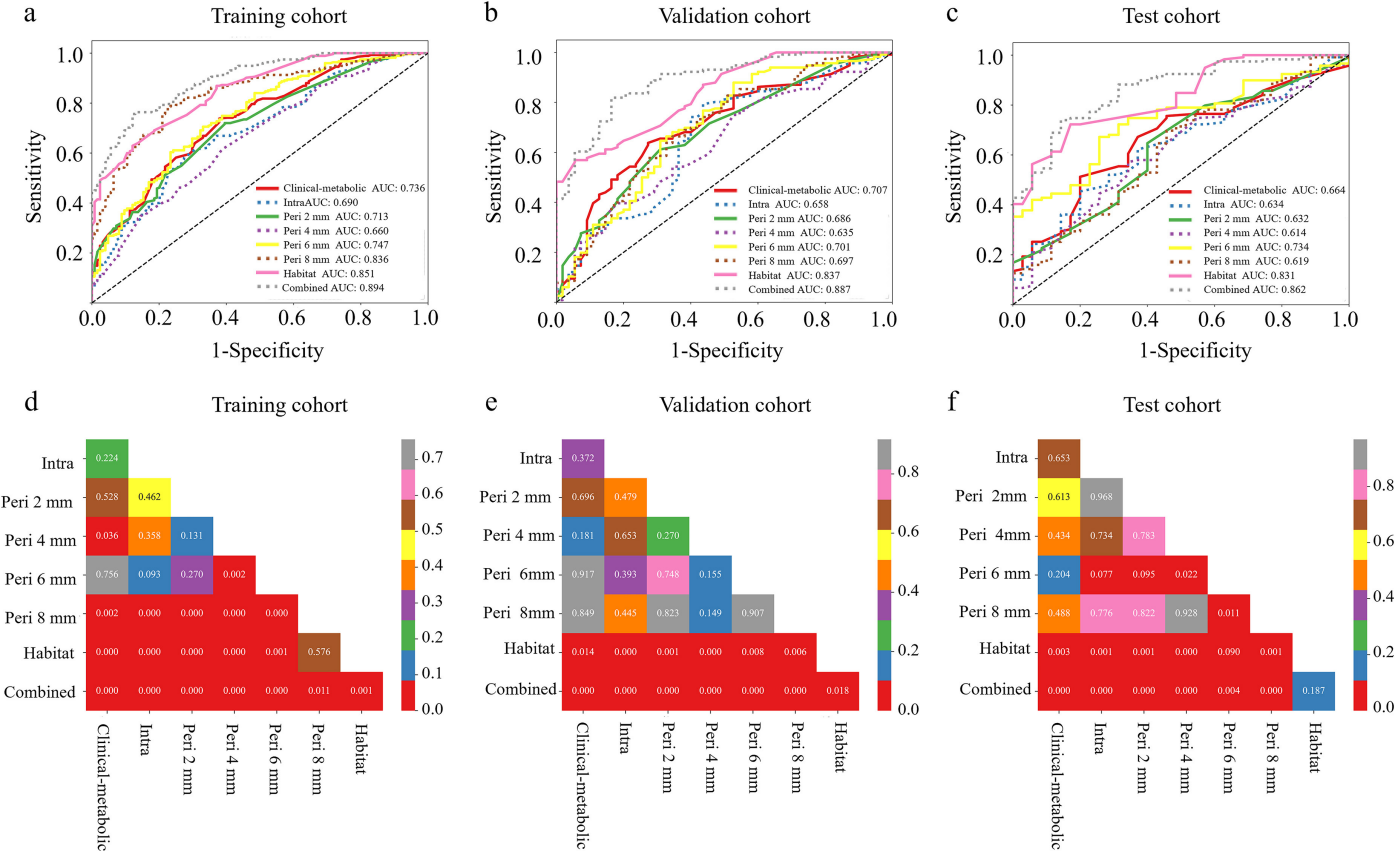
ROC curves and DeLong test comparisons of different optimal machine learning models in the training, validation and test cohorts. Panels **(a–c)** show ROC curves; panels **(d–f)** present DeLong test results.

Using the optimal machine learning algorithm, a combined model was constructed integrating clinical-metabolic, intratumoral, optimal 6 mm peritumoral, and habitat features. The model demonstrated strong performance in both the training (AUC = 0.894, 95% CI: 0.86–0.93) and validation cohorts (AUC = 0.887, 95% CI: 0.84–0.94), with corresponding PPVs of 0.933 and 0.945; these outcomes surpassed all individual component models (all *P* < 0.05) (Table 4). In the test cohort, the combined model achieved an AUC of 0.862 (95% CI: 0.80–0.93), with a sensitivity of 0.739, specificity of 0.857, and PPV of 0.946, significantly outperforming the clinical-metabolic, intratumoral, and other peritumoral models (all *P* < 0.05) while demonstrating comparable efficacy to the habitat model (*P* = 0.187). The habitat model itself also significantly exceeded the performance of other individual models (all *P* < 0.05) (Supplementary Table 4 and Figure 5).

**TABLE 4:** Diagnostic performance of the combined model for EGFR mutation detection.

Dataset	AUC	95% CI	ACC	Sen	Spe	PPV	NPV
Training	0.894	0.863–0.925	0.794	0.756	0.879	0.933	0.619
Validation	0.887	0.837–0.938	0.825	0.819	0.836	0.913	0.687
Test	0.862	0.798–0.926	0.766	0.739	0.857	0.946	0.492

AUC, area under the curve; 95% CI, 95% confidence interval; ACC, accuracy; Sen, sensitivity; Spe, specificity; PPV, positive predictive value; NPV, negative predictive value.

^1^ www.itksnap.org

Comparative evaluation confirmed the 6 mm peritumoral model as the optimal peritumoral signature (test cohort AUC = 0.734, specificity = 0.743, PPV = 0.899), outperforming both the 4 and 8 mm peritumoral models (all *P* < 0.05) (Figure 5).

Calibration analysis indicated strong concordance between predicted and actual outcomes (*P* > 0.05), supporting reliable generalizability (Supplementary Figures 3a–c). DCA further validated the superior net clinical benefit of the combined model (Supplementary Figures 3d–f). A visual nomogram, weighted primarily by habitat and 6 mm peritumoral features, was established to predict EGFRm status, facilitating clinical decision-making and prognostic assessment (Supplementary Figure 3g).

### Habitat model SHAP visualization

**FIGURE 6:**
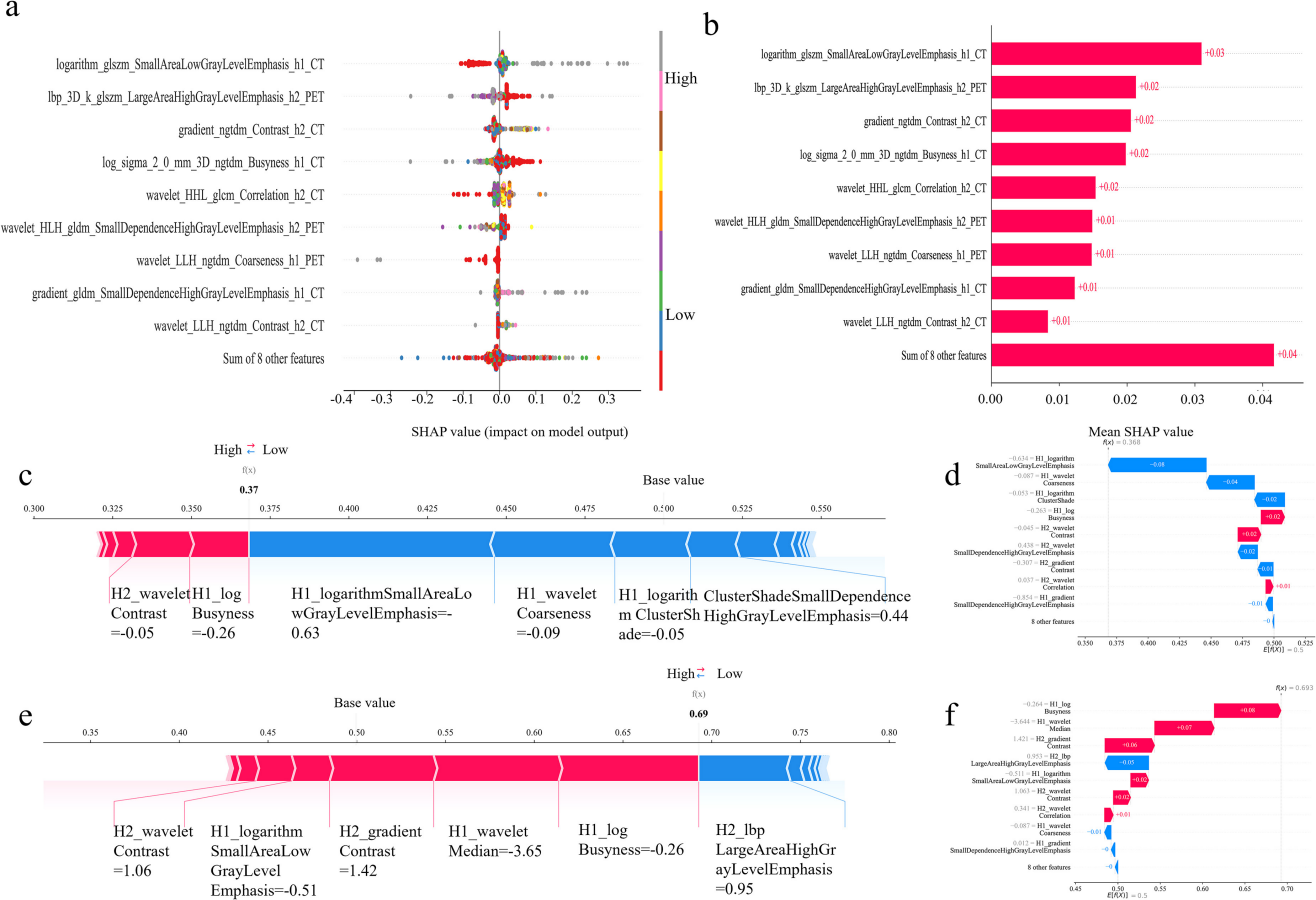
Integrated SHAP analysis of the habitat model for EGFR mutation status prediction. **(a)** Summary plot showing the global distribution of feature importance across the entire dataset. **(b)** Bar plot illustrating the quantitative contribution of individual features to model decision-making. **(c, e)** Force plots demonstrate how feature-specific SHAP values shift predictions toward EGFRwt or EGFRm status. **(d, f)** Waterfall plots present the cumulative predictive effects of the most influential features. Panels **(c,d)** show a representative EGFRwt case (habitat model: SHAP = 0.37, predicted EGFRwt); panels **(e, f)** show an EGFRm case (habitat model: SHAP = 0.69, predicted EGFRm).

SHAP analysis was applied to enhance interpretability of the ExtraTrees habitat model. The summary and bar plots (Figures 6a,b) highlighted the dominant contribution of habitat subregions H1 and H2, encompassing 16 of the 17 included features. Within EGFRm prediction, CT-derived features exhibited greater importance than PET-derived features, with CT features representing 6 of the top 9 core features (67%). Texture features emerged as the principal contributors, with Gray-Level Size Zone Matrix (GLSZM) and Neighborhood Gray-Tone Difference Matrix (NGTDM)-related features ranking among the top four. Force and waterfall plots demonstrated variations in the contributions of individual features and samples to prediction outcomes (Figures 6c–f). Overall, intratumoral habitats, particularly subregion H2, acted as key drivers of model predictions, suggesting that this subregion is enriched with core imaging biomarkers critical for accurate EGFRm prediction.

## Discussion

This study aimed to assess the predictive efficacy of ^18^F-FDG PET/CT-based combined models in determining EGFRm status in patients with lung adenocarcinoma. The results demonstrated that the tumor habitat model achieved markedly superior predictive performance compared with other machine learning approaches and the clinical-metabolic model, while the peritumoral 6 mm model exhibited optimal diagnostic accuracy. Moreover, the integrated model incorporating multi-dimensional features achieved favorable and numerically optimal predictive performance. Interpretability and visualization of the habitat model were further enhanced using the SHAP method.

Habitat analysis enables detailed characterization of intratumoral heterogeneity, quantification of tumor microenvironmental attributes, and identification of potential imaging biomarkers. Previous studies have established the utility of habitat radiomics in evaluating treatment response, predicting microvascular invasion, and supporting prognostic assessment (18, 26–29). However, limited research has applied this methodology to predict EGFRm status in lung cancer. In the present study, the k-means-based habitat model achieved an external test set AUC of 0.83 (95% CI: 0.76–0.90), outperforming conventional models. Prior work by Wu et al. (30) reported an external validation AUC of 0.790 for EGFRm prediction using habitat analysis, while Yang et al. (31) further validated its potential in a multicenter study involving 293 NSCLC patients with brain metastases. In their external validation cohort, AUCs for predicting overall EGFRm, Exon-19 mutation, and Exon-21 mutation were 0.812 (95% CI: 0.68–0.95), 0.818 (95% CI: 0.61–1.00), and 0.800 (95% CI: 0.60–1.00), respectively. These findings are consistent with the current results, reinforcing the clinical relevance of habitat radiomics. The integration of multi-omics data may further enhance predictive accuracy and facilitate personalized therapeutic strategies.

SHAP analysis revealed that habitat subregions H1 and H2, along with their associated texture features, served as the primary predictors. Of the 17 significant features identified, 16 were localized within these two subregions. Additionally, CT-derived features demonstrated superior predictive performance compared with PET-derived features, with GLSZM and NGTDM texture features emerging as the predominant contributors. GLSZM captures the spatial distribution of homogeneous gray-level regions, whereas NGTDM quantifies local contrast (32), aligning with findings from prior studies (5, 33). Specifically, Aide et al. (5) reported that the NGTDM-derived Busyness feature could predict tumor response in pediatric soft tissue sarcoma, while GLSZM features independently predicted progression-free survival. In a quantitative MRI study (34), NGTDM was the sole texture family in which most features exhibited excellent reproducibility (ICC > 0.90) within a specific MRI sequence (MRF T1). Similarly, GLSZM demonstrated favorable reproducibility in certain sequences (e.g., T1-weighted MPRAGE). These observations indicate that the key GLSZM and NGTDM features selected by the current model possess both robust predictive capacity and stability for clinical application (35).

The multidimensional integrated model developed in this study, incorporating tumor habitat characteristics, intratumoral and peritumoral radiomic features, and clinical-metabolic parameters, demonstrated favorable and robust predictive performance for EGFRm in lung adenocarcinoma. The model achieved an AUC of 0.86 (95% CI: 0.80–0.93) in external validation, significantly outperforming single-feature models (*P* < 0.05). These results highlight the enhanced predictive capability of multimodal data integration relative to unimodal approaches. Consistent with these findings, prior studies on EGFRm prediction have reported similar outcomes: a dual-energy CT radiomics-clinical feature model attained a validation AUC of 0.80 (95% CI: 0.68–0.94) (36), whereas a PET/CT-clinical feature model in NSCLC patients achieved a test AUC of 0.806 (95% CI: 0.67–0.95) (37). Furthermore, Zhang et al. (38) developed an SVM-based radiomics-clinical feature model, yielding a validation AUC of 0.827 and an accuracy of 0.753, further corroborating the superiority of combined models over single-modality approaches. Notably, the integrated model in the present study demonstrated improved performance, with a validation AUC of 0.862 and accuracy of 0.766, highlighting the efficacy of the multimodal integration strategy. These findings validate the advantages of comprehensive feature fusion and emphasize the clinical significance of this approach in predicting EGFRm in lung adenocarcinoma.

The peritumoral region exhibits considerable potential for EGFRm prediction. In this study, peritumoral models were constructed with varying expansion distances (2, 4, 6, and 8 mm) to evaluate their predictive performance. The results indicated that model efficacy increased with expanding peritumoral range, reaching optimal performance at 6 mm (AUC: 0.693; 95% CI: 0.598–0.788). Beyond this distance, further expansion led to reduced performance, likely due to interference from increased background noise. Previous research has similarly highlighted the value of peritumoral radiomics, although the reported optimal ranges vary: a CT-based analysis of 779 lung adenocarcinoma cases (39) identified a 4 mm peritumoral region as providing incremental predictive value (AUC: 0.635; 95% CI: 0.540–0.731), whereas Wu et al. (30) reported optimal performance with a 3 mm peritumoral model (AUC: 0.684; 95% CI: 0.541–0.828) in a cohort of 438 early-stage NSCLC patients. These discrepancies suggest the critical importance of selecting an appropriate peritumoral range, which, as indicated in a systematic review (10), depends on the specific methodology employed for region-of-interest segmentation. Future studies should focus on optimizing peritumoral segmentation protocols and feature extraction techniques to enhance the clinical utility of predictive models.

Conventional metabolic parameters derived from ^18^F-FDG PET/CT, including maximum standardized uptake value (SUV_*max*_), MTV, and TLG, possess substantial diagnostic and prognostic value for assessing EGFRm status. The present findings indicate that reduced SUV_*max*_ serves as an independent predictor of EGFRm (OR = 0.94, 95% CI: 0.91–0.97; *P* = 0.001), whereas neither MTV (OR = 1.28, 95% CI: 1.07–1.52; *P* = 0.106) nor TLG (OR = 1.00, 95% CI: 1.00–1.00; *P* = 0.734) demonstrated significant predictive utility, consistent with previous reports (40, 41). The underlying molecular mechanisms may involve EGFRm-mediated activation of glutamine metabolism, downregulation of glycolysis-related genes, and suppression of glucose metabolism via the MEK/ERK/ELK1 signaling pathway (42, 43). Additionally, EGFRm modulates cellular proliferation and DNA damage repair through the PI3K-Akt-mTOR axis (44, 45), potentially accounting for the observed hypometabolic phenotype. These observations are further supported by Ni et al. (46), who reported that MTV and TLG, primarily reflecting tumor burden rather than metabolic activity, were not independent predictors of EGFRm status.

Baseline clinical characteristics revealed a higher prevalence of EGFRm among younger, female, and non-smoking/non-drinking patients with lung adenocarcinoma. Multivariate analysis confirmed that female sex remained an independent predictor of EGFRm (OR = 2.71, 95% CI: 1.83–4.02; *P* < 0.001) after adjustment for potential confounders. These findings align with previous literature reporting elevated EGFRm rates among young, female non-smokers (47–49) and are further corroborated by studies identifying female sex as an independent predictor after accounting for smoking history (50). The observed association may involve estrogen-related pathways, although the precise mechanisms warrant further investigation.

Several limitations of this study should be acknowledged. First, the patient cohort was recruited from two centers with notable differences in baseline characteristics (Cyfra 21-1, NSE levels, TNM stage, and imaging features) and laboratory protocols, likely reflecting the disparate stage distributions between centers (42.8% stage I–II in Center 1 vs. 78.57% stage III–IV in Center 2). Second, although the predictive performance of the radiomics-based model was assessed, deep learning algorithms were not incorporated. Future research should explore the integration of artificial intelligence techniques to enhance model accuracy and generalizability. Third, EGFRm testing followed local laboratory standards rather than unified cross-center protocols, and no analytical harmonization was performed between NGS and PCR assays, which may introduce methodological heterogeneity. Fourth, this study did not include EGFR subtype analysis (e.g., Exon 19 vs. Exon 21), potentially limiting the model’s ability to predict specific mutation subtypes, nor did it evaluate deep learning-based approaches, which may provide additional predictive power and robustness.

In conclusion, radiomic analysis derived from primary tumor ^18^F-FDG PET/CT demonstrates significant predictive value for EGFRm status in lung adenocarcinoma. Both the habitat and combined models exhibit robust and favorable predictive performance compared with conventional single-dimensional models, providing reliable imaging biomarkers to support personalized therapeutic decision-making.

## Data Availability

The datasets presented in this study can be found in online repositories. The names of the repository/repositories and accession number(s) can be found in this article/Supplementary material.
