# Supplementary material for: The predictive value of 18F-FDG PET/CT habitat radiomics combined model in evaluating EGFR gene mutations in lung adenocarcinoma
Source: Front Med (Lausanne). 2026 Jun 12;13:1868229. doi: 10.3389/fmed.2026.1868229 (PMC13303213; doi:10.3389/fmed.2026.1868229)
Supplement: Supplementary file 1 [file Data_Sheet_1.docx]

**Supplementary Materials**

1. **Supplementary Table 1**
2. **Supplementary Table 2**
3. **Supplementary Table 3**
4. **Supplementary Table 4**
5. **Supplementary Table 5**
6. **Supplementary Figure 1**
7. **Supplementary Figure 2**
8. **Supplementary Figure 3**

**Supplementary Table 1.** Technical Parameters for Different PET/CT models.

| **PET/CT Model** | **Kilovoltage peak(kVp)** | **Milliampere-seconds (mAs)** | **Crystal size (LYSO)** | **Reconstruction method** | **PET slice thickness** | **PET Pixel Spacing** | **CT slice thickness** | **CT Pixel Spacing** |
| --- | --- | --- | --- | --- | --- | --- | --- | --- |
| Gemini GXL | 120 | 40 | 4 mm × 5 mm × 20 mm | Line of Response (LOR) | 4 mm | 4mm×4 mm | 2.5 mm | 1 mm × 1 mm |
| Biograph 16 PET/CT | 120 | 150 | 4 mm × 4 mm × 20 mm | OSEM | 5mm | 4mm×4 mm | 1 mm | 1 mm × 1 mm |

**Supplementary Table 2.** Thirty-nine parameters for local habitat characterization

| **Features** | **Features** | **Features** |
| --- | --- | --- |
| log_firstorder_Entropy | original_firstorder_Entropy | original_ngtdm_Strength |
| log_firstorder_MeanAbsoluteDeviation | original_glcm_InverseVariance | wavelet_firstorder_MeanAbsoluteDeviation |
| log_firstorder_Median | original_firstorder_Median | wavelet_firstorder_Median |
| log_glcm_DifferenceAverage | original_glcm_DifferenceAverage | wavelet_glcm_DifferenceAverage |
| log_glcm_DifferenceEntropy | original_glcm_DifferenceEntropy | wavelet_glcm_DifferenceEntropy |
| log_glcm_DifferenceVariance | original_glcm_DifferenceVariance | wavelet_glcm_DifferenceVariance |
| log_glcm_Imc1 | original_glcm_Imc1 | wavelet_glcm_Imc1 |
| log_glcm_InverseVariance | original_firstorder_Entropy | wavelet_glcm_InverseVariance |
| log_glcm_JointEnergy | original_glcm_JointEnergy | wavelet_glcm_JointEnergy |
| log_glcm_JointEntropy | original_glcm_JointEntropy | wavelet_glcm_JointEntropy |
| log_glcm_SumEntropy | original_glcm_JointEntropy | wavelet_glcm_SumEntropy |
| log_ngtdm_Contrast | original_ngtdm_Contrast | wavelet_ngtdm_Contrast |
| log_ngtdm_Strength | original_ngtdm_Strength | wavelet_ngtdm_Strength |

**Supplementary Table 3.** Inter-group consistency results of VOI masks in 50 patients

| **No.** | **DICE coefficient** | **No.** | **DICE coefficient** | **No.** | **DICE coefficient** | **No.** | **DICE coefficient** | **No.** | **DICE coefficient** |
| --- | --- | --- | --- | --- | --- | --- | --- | --- | --- |
| 1 | 0.974 | 11 | 0.976 | 21 | 0.981 | 31 | 0.961 | 41 | 0.951 |
| 2 | 0.971 | 12 | 0.904 | 22 | 0.976 | 32 | 0.983 | 42 | 0.947 |
| 3 | 0.991 | 13 | 0.923 | 23 | 0.996 | 33 | 0.982 | 43 | 0.984 |
| 4 | 0.979 | 14 | 0.977 | 24 | 0.986 | 34 | 0.909 | 44 | 0.976 |
| 5 | 0.977 | 15 | 0.981 | 25 | 0.979 | 35 | 0.982 | 45 | 0.984 |
| 6 | 0.989 | 16 | 0.975 | 26 | 0.965 | 36 | 0.975 | 46 | 0.986 |
| 7 | 0.898 | 17 | 0.899 | 27 | 0.964 | 37 | 0.965 | 47 | 0.986 |
| 8 | 0.913 | 18 | 0.964 | 28 | 0.983 | 38 | 0.978 | 48 | 0.983 |
| 9 | 0.984 | 19 | 0.996 | 29 | 0.983 | 39 | 0.933 | 49 | 0.988 |
| 10 | 0.977 | 20 | 0.990 | 30 | 0.987 | 40 | 0.984 | 50 | 0.959 |

**Supplementary Table 4. Comparative performance analysis of diagnostic models for EGFR mutation detection**

| **Model** | **Machine Learning Algorithm** | **Dataset** | **AUC** | **95%CI** | **ACC** | **Sen** | **Spe** | **PPV** | **NPV** |
| --- | --- | --- | --- | --- | --- | --- | --- | --- | --- |
| Clinical-metabolic model | SVM | Training | 0.576 | 0.514 - 0.639 | 0.539 | 0.542 | 0.532 | 0.720 | 0.344 |
|  |  | Validation | 0.521 | 0.424 - 0.619 | 0.532 | 0.526 | 0.545 | 0.709 | 0.353 |
|  |  | Test | 0.501 | 0.392 - 0.610 | 0.526 | 0.471 | 0.714 | 0.848 | 0.284 |
|  | RF | Training | 0.736 | 0.684 - 0.788 | 0.697 | 0.742 | 0.597 | 0.803 | 0.510 |
|  |  | Validation | 0.707 | 0.624 - 0.790 | 0.661 | 0.681 | 0.618 | 0.790 | 0.479 |
|  |  | Test | 0.664 | 0.567 - 0.761 | 0.461 | 0.353 | 0.829 | 0.875 | 0.274 |
|  | ExtraTrees | Training | 0.708 | 0.652 - 0.763 | 0.659 | 0.687 | 0.597 | 0.791 | 0.462 |
|  |  | Validation | 0.680 | 0.595 - 0.765 | 0.684 | 0.698 | 0.655 | 0.810 | 0.507 |
|  |  | Test | 0.647 | 0.539 - 0.754 | 0.481 | 0.412 | 0.714 | 0.831 | 0.263 |
| Intratumoral model | SVM | Training | 0.588 | 0.530 - 0.647 | 0.569 | 0.545 | 0.621 | 0.761 | 0.381 |
|  |  | Validation | 0.567 | 0.474 - 0.659 | 0.573 | 0.526 | 0.673 | 0.772 | 0.402 |
|  |  | Test | 0.551 | 0.443 - 0.658 | 0.558 | 0.546 | 0.600 | 0.823 | 0.280 |
|  | RF | Training | 0.805 | 0.761 - 0.848 | 0.744 | 0.760 | 0.710 | 0.853 | 0.571 |
|  |  | Validation | 0.638 | 0.548 - 0.729 | 0.673 | 0.716 | 0.582 | 0.783 | 0.492 |
|  |  | Test | 0.563 | 0.452 - 0.674 | 0.799 | 0.992 | 0.143 | 0.797 | 0.833 |
|  | ExtraTrees | Training | 0.690 | 0.635 - 0.745 | 0.639 | 0.615 | 0.694 | 0.816 | 0.448 |
|  |  | Validation | 0.658 | 0.567 - 0.748 | 0.719 | 0.793 | 0.564 | 0.793 | 0.564 |
|  |  | Test | 0.634 | 0.536 - 0.733 | 0.532 | 0.454 | 0.800 | 0.885 | 0.301 |
| Habitat model | SVM | Training | 0.741 | 0.691 - 0.792 | 0.624 | 0.531 | 0.831 | 0.874 | 0.444 |
|  |  | Validation | 0.675 | 0.588 - 0.761 | 0.678 | 0.733 | 0.564 | 0.780 | 0.500 |
|  |  | Test | 0.688 | 0.597 - 0.778 | 0.584 | 0.529 | 0.771 | 0.887 | 0.325 |
|  | RF | Training | 0.876 | 0.841 - 0.912 | 0.774 | 0.727 | 0.879 | 0.930 | 0.592 |
|  |  | Validation | 0.668 | 0.586 - 0.750 | 0.573 | 0.422 | 0.891 | 0.891 | 0.422 |
|  |  | Test | 0.678 | 0.577 - 0.779 | 0.649 | 0.647 | 0.657 | 0.865 | 0.354 |
|  | ExtraTrees | Training | 0.851 | 0.814 - 0.889 | 0.707 | 0.629 | 0.879 | 0.920 | 0.517 |
|  |  | Validation | 0.837 | 0.777 - 0.896 | 0.690 | 0.569 | 0.945 | 0.957 | 0.510 |
|  |  | Test | 0.831 | 0.761 - 0.900 | 0.747 | 0.723 | 0.829 | 0.935 | 0.468 |

SVM: Support Vector Machine; RF: Random Forest; ExtraTrees: Extremely Randomized Trees; AUC: Area Under the Curve; 95% CI: 95% Confidence Interval; ACC: Accuracy; Sen: Sensitivity; Spe: Specificity; PPV: Positive Predictive Value; NPV: Negative Predictive Value

**Supplementary Table 5. Performance Analysis of Different Peritumoral Models Constructed by Three Machine Learning Algorithms for EGFR Mutation Diagnosis**

| **Model** | **Machine Learning Algorithm** | **Dataset** | **AUC** | **95%CI** | | **ACC** | | **Sen** | | **Spe** | | **PPV** | | **NPV** | |  |
| --- | --- | --- | --- | --- | --- | --- | --- | --- | --- | --- | --- | --- | --- | --- | --- | --- |
| Peritumoral  2 mm model | SVM | Training | 0.618 | 0.559 - 0.678 | | 0.591 | | 0.575 | | 0.629 | | 0.775 | | 0.400 | |  |
|  |  | Validation | 0.657 | 0.568 - 0.745 | | 0.696 | | 0.750 | | 0.582 | | 0.791 | | 0.525 | |  |
|  |  | Test | 0.501 | 0.391 - 0.611 | | 0.364 | | 0.218 | | 0.857 | | 0.839 | | 0.244 | |  |
|  | RF | Training | 0.713 | 0.660 - 0.765 | | 0.684 | | 0.720 | | 0.605 | | 0.802 | | 0.493 | |  |
|  |  | Validation | 0.686 | 0.603 - 0.769 | | 0.637 | | 0.612 | | 0.691 | | 0.807 | | 0.458 | |  |
|  |  | Test | 0.632 | 0.529 - 0.735 | | 0.636 | | 0.647 | | 0.600 | | 0.846 | | 0.333 | |  |
|  | ExtraTrees | Training | 0.700 | 0.645 - 0.754 | | 0.637 | | 0.578 | | 0.766 | | 0.846 | | 0.450 | |  |
|  |  | Validation | 0.678 | 0.592 - 0.763 | | 0.684 | | 0.733 | | 0.582 | | 0.787 | | 0.508 | |  |
|  |  | Test | 0.606 | 0.503 - 0.709 | | 0.623 | | 0.630 | | 0.600 | | 0.843 | | 0.323 | |  |
| Peritumoral  4 mm model | SVM | Training | 0.597 | 0.536 - 0.658 | | 0.674 | | 0.829 | | 0.331 | | 0.733 | | 0.466 | |  |
|  |  | Validation | 0.621 | 0.530 - 0.713 | | 0.626 | | 0.629 | | 0.618 | | 0.777 | | 0.442 | |  |
|  |  | Test | 0.631 | 0.533 - 0.728 | | 0.636 | | 0.622 | | 0.686 | | 0.871 | | 0.348 | |  |
|  | RF | Training | 0.643 | 0.586 - 0.700 | | 0.534 | | 0.415 | | 0.798 | | 0.820 | | 0.381 | |  |
|  |  | Validation | 0.632 | 0.541 - 0.723 | | 0.673 | | 0.776 | | 0.455 | | 0.750 | | 0.490 | |  |
|  |  | Test | 0.620 | 0.520 - 0.721 | | 0.597 | | 0.580 | | 0.657 | | 0.852 | | 0.315 | |  |
|  | ExtraTrees | Training | 0.660 | 0.603 - 0.717 | | 0.614 | | 0.615 | | 0.613 | | 0.779 | | 0.418 | |  |
|  |  | Validation | 0.635 | 0.547 - 0.723 | | 0.661 | | 0.750 | | 0.473 | | 0.750 | | 0.473 | |  |
|  |  | Test | 0.614 | 0.512 - 0.716 | | 0.591 | | 0.571 | | 0.657 | | 0.850 | | 0.311 | |  |
| Peritumoral  6 mm model | SVM | Training | 0.722 | 0.670 - 0.775 | | 0.679 | | 0.702 | | 0.629 | | 0.808 | | 0.487 | |  |
|  |  | Validation | 0.644 | 0.559 - 0.729 | | 0.544 | | 0.388 | | 0.873 | | 0.865 | | 0.403 | |  |
|  |  | Test | 0.629 | 0.515 - 0.743 | | 0.714 | | 0.782 | | 0.486 | | 0.838 | | 0.395 | |  |
|  | RF | Training | 0.747 | 0.695 - 0.799 | | 0.694 | | 0.705 | | 0.669 | | 0.826 | | 0.506 | |  |
|  |  | Validation | 0.701 | 0.613 - 0.789 | | 0.749 | | 0.879 | | 0.473 | | 0.779 | | 0.650 | |  |
|  |  | Test | 0.734 | 0.650 - 0.817 | | 0.688 | | 0.672 | | 0.743 | | 0.899 | | 0.400 | |  |
|  | ExtraTrees | Training | 0.759 | 0.710 - 0.809 | | 0.689 | | 0.691 | | 0.685 | | 0.830 | | 0.500 | |  |
|  |  | Validation | 0.660 | 0.572 - 0.748 | | 0.637 | | 0.638 | | 0.636 | | 0.787 | | 0.455 | |  |
|  |  | Test | 0.693 | 0.598 - 0.788 | | 0.727 | | 0.773 | | 0.571 | | 0.860 | | 0.426 | |  |
| Peritumoral  8 mm model | SVM | Training | 0.648 | 0.589 - 0.706 | | 0.659 | | 0.724 | | 0.516 | | 0.768 | | 0.457 | |  |
|  |  | Validation | 0.629 | 0.542 - 0.716 | | 0.573 | | 0.474 | | 0.782 | | 0.821 | | 0.413 | |  |
|  |  | Test | 0.582 | | 0.478 - 0.687 | | 0.565 | | 0.529 | | 0.686 | | 0.851 | | 0.300 | |
|  | RF | Training | 0.836 | 0.795 - 0.876 | | 0.782 | | 0.785 | | 0.774 | | 0.885 | | 0.619 | |  |
|  |  | Validation | 0.697 | 0.611 - 0.783 | | 0.719 | | 0.828 | | 0.491 | | 0.774 | | 0.574 | |  |
|  |  | Test | 0.619 | 0.513 - 0.726 | | 0.662 | | 0.697 | | 0.543 | | 0.838 | | 0.345 | |  |
|  | ExtraTrees | Training | 0.748 | 0.698 - 0.799 | | 0.687 | | 0.684 | | 0.694 | | 0.832 | | 0.497 | |  |
|  |  | Validation | 0.682 | 0.599 - 0.766 | | 0.591 | | 0.466 | | 0.855 | | 0.871 | | 0.431 | |  |
|  |  | Test | 0.668 | 0.566 - 0.770 | | 0.604 | | 0.555 | | 0.771 | | 0.892 | | 0.337 | |  |

SVM: Support Vector Machine; RF: Random Forest; ExtraTrees: Extremely Randomized Trees; AUC: Area Under the Curve; 95% CI: 95% Confidence Interval; ACC: Accuracy; Sen: Sensitivity; Spe: Specificity; PPV: Positive Predictive Value; NPV: Negative Predictive Value


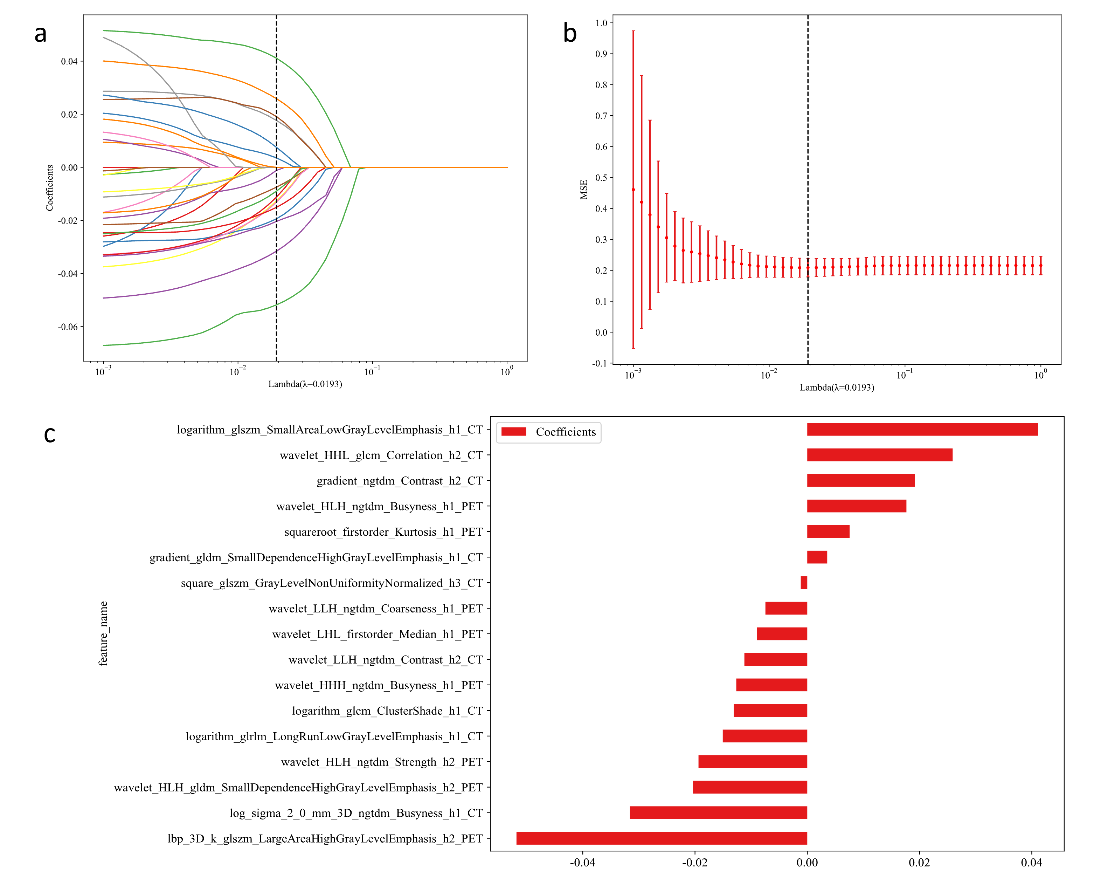


**Supplementary Figure 1.** Feature selection process for the habitat LASSO regression model. (a) Coefficient shrinkage trajectory of the regularization parameter (λ). (b) Mean squared error (MSE) curve derived from 10-fold cross-validation. (c) Histogram of the final selected features. LASSO: Least Absolute Shrinkage and Selection Operator; MSE: Mean Squared Error.


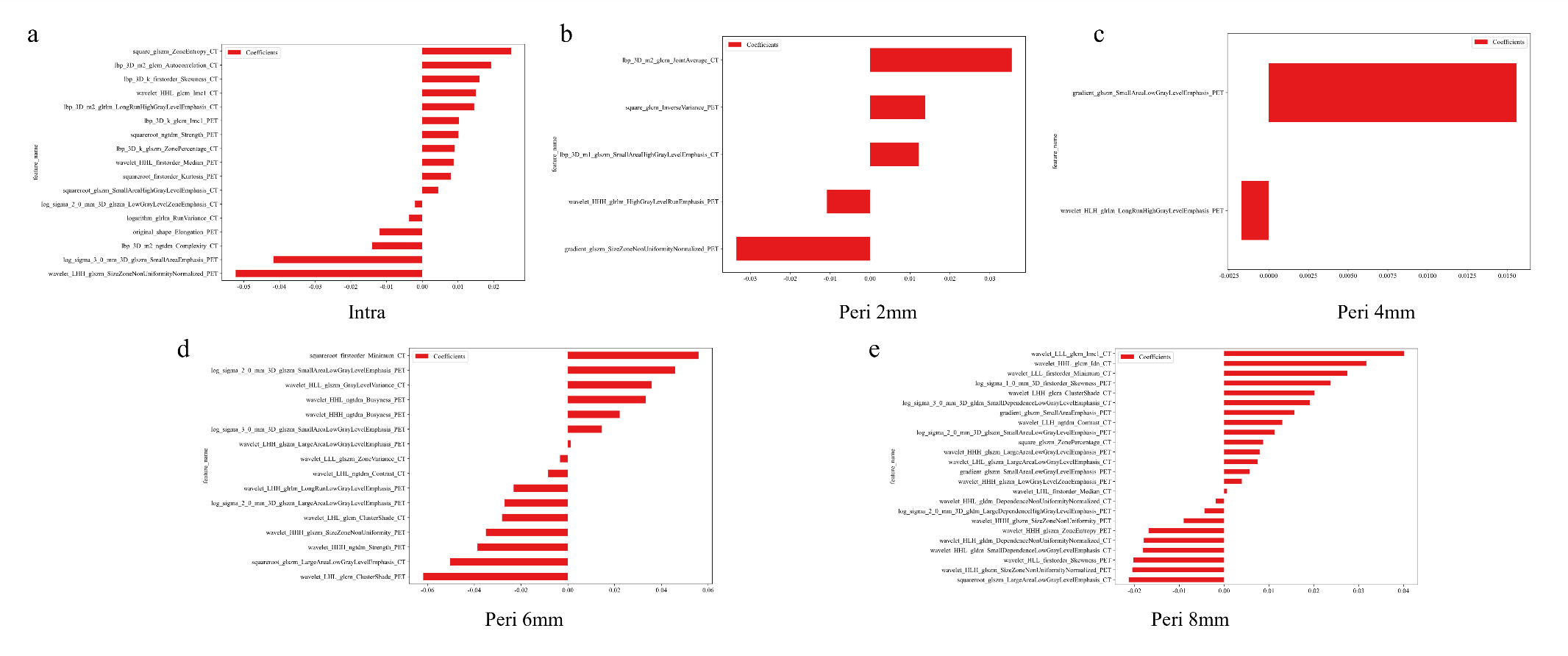


Supplementary Figure 2. Radiomic feature selection for intratumoral and peritumoral (2 mm/4 mm/6 mm/8 mm) model construction.


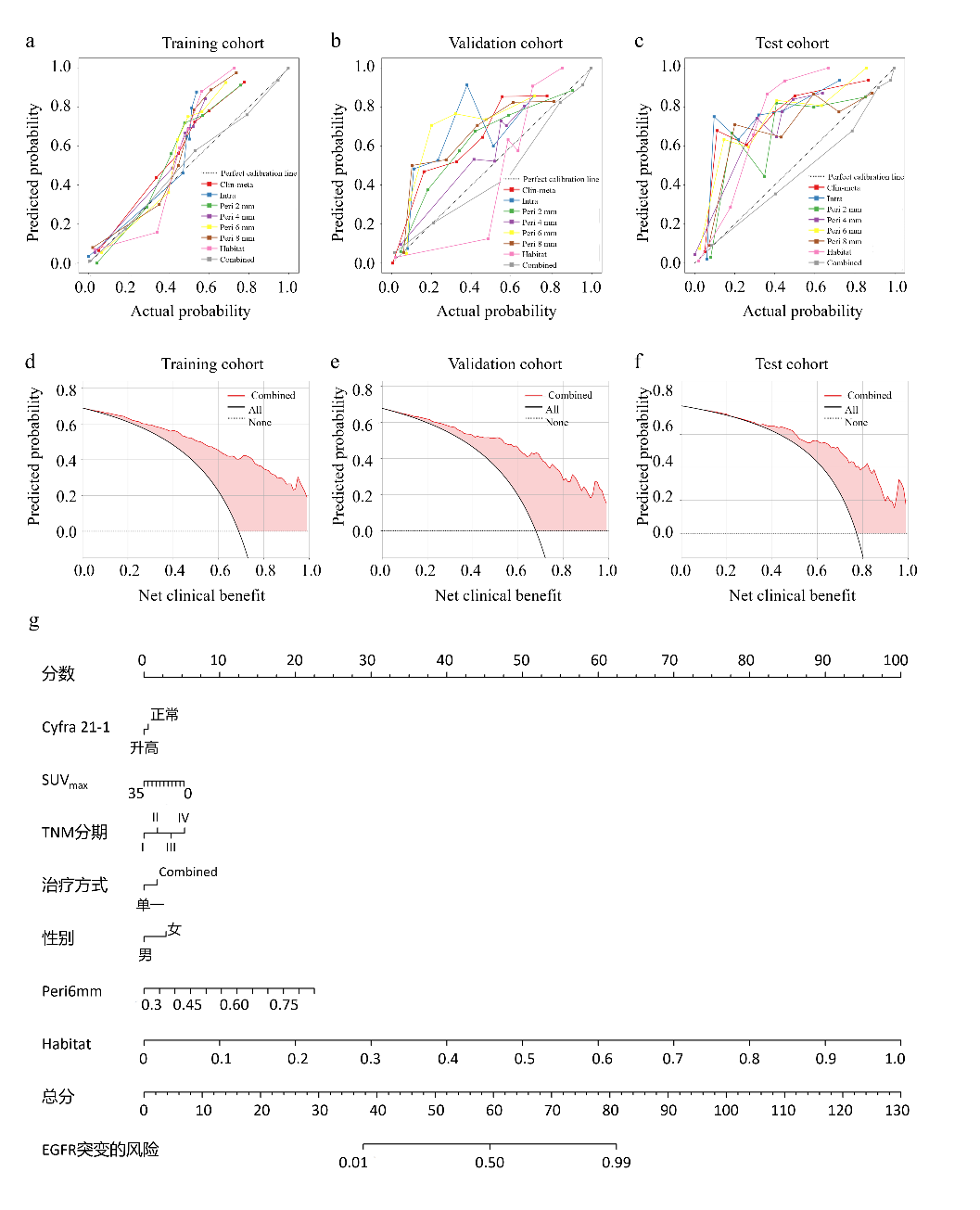


Supplementary Figure 3. Calibration curves, decision curve analysis (DCA) plots, and nomogram of the combined predictive model. Subfigures (a) – (c) show calibration curves for the training, validation, and test cohorts; subfigures (d) – (f) present DCA plots; subfigure (g) shows the nomogram representing the combined predictive model developed using the training dataset.
